# Supplementary material for: 3D Brain Vascular Niche Model Captures Glioblastoma Infiltration, Dormancy, and Gene Signatures
Source: Adv Sci (Weinh). 2025 Jun 19;12(33):e00689. doi: 10.1002/advs.202500689 (PMC12412466; doi:10.1002/advs.202500689)
Supplement: Supplementary file 1 — Supporting Information [file ADVS-12-e00689-s002.docx]

**
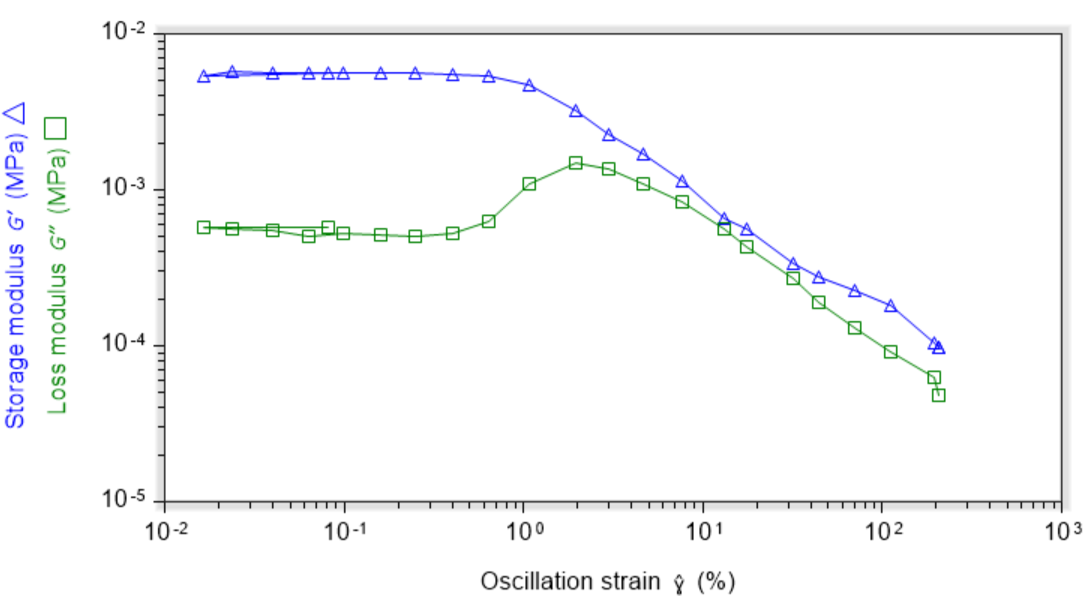
**

**Figure S1. Rheological Characterization of Hydrogel Stiffness.**

Amplitude sweep of hydrogel sample showing storage modulus (G′) and loss modulus (G″) as a function of strain. The sweep was performed from 0.01% to 200% strain at a constant angular frequency, revealing the linear viscoelastic region (LVER) between 0.1% and 1% strain, where G′ remains constant.

**
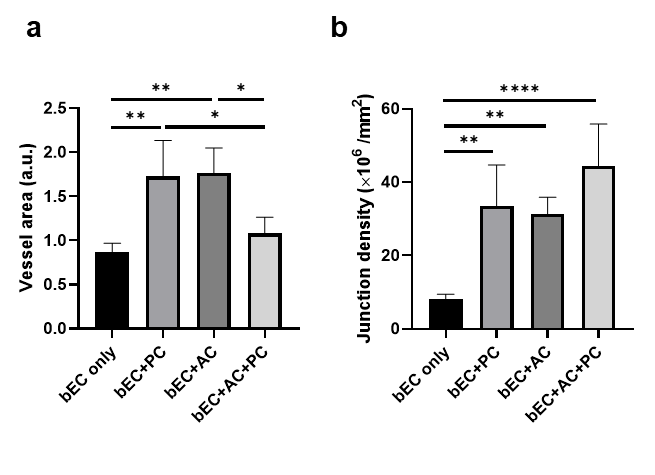
**

**Figure S2.** **Optimization of astrocyte and pericyte seeding densities for vascular formation in the 3D model.**

(a) Total vessel area and (b) junction density were quantified in 3D cultures across different seeding concentrations of pericytes (PC) and astrocytes (AC). n = 4–6 per condition. Statistical analysis was performed using one-way ANOVA with Tukey's post hoc test. ^*^ *P* ≤ 0.05, ^**^ *P* ≤ 0.01, ^***^ *P* ≤ 0.001, ^****^ *P* ≤ 0.0001.

**
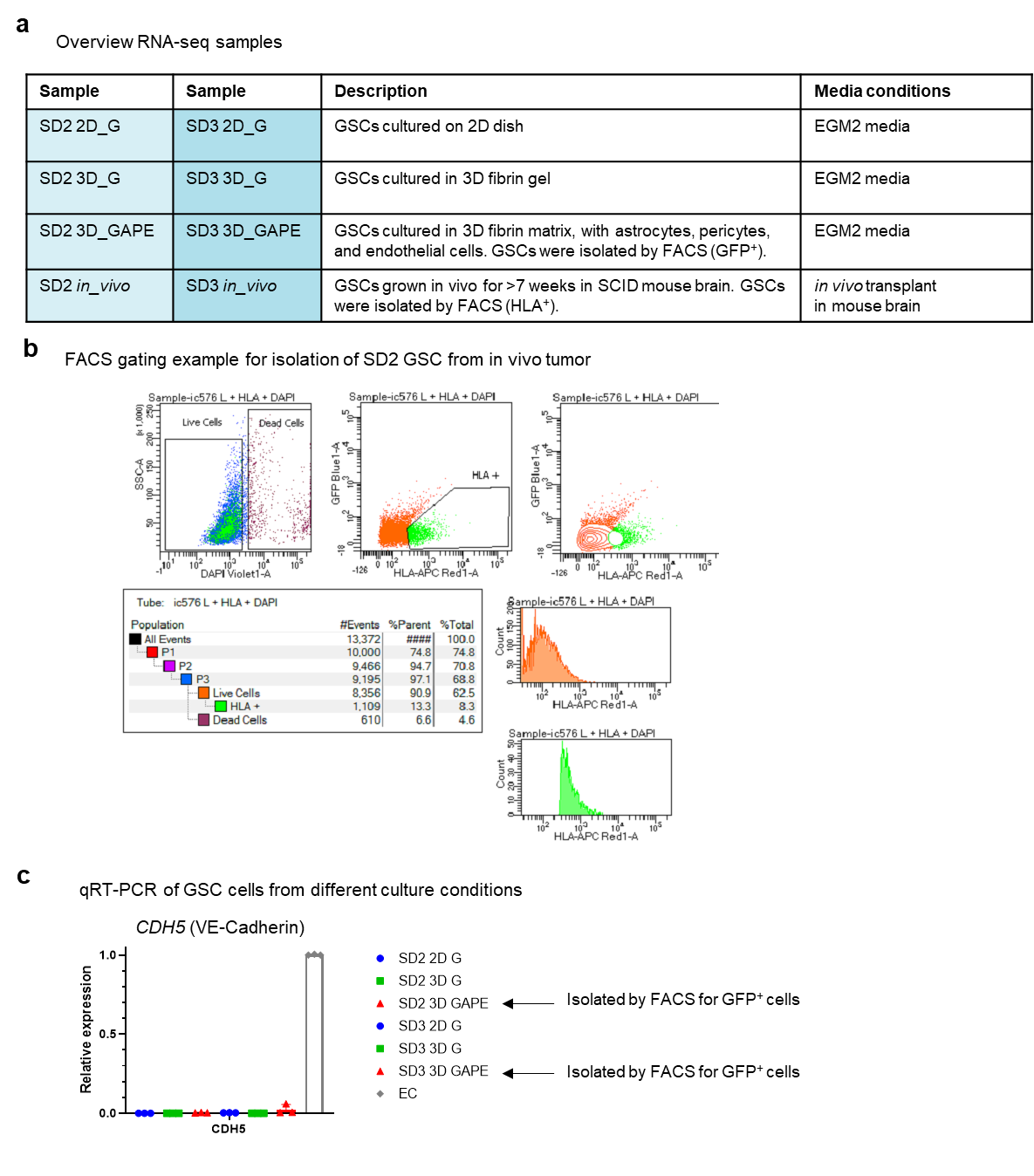
**

**Figure S3. Collection of RNA-seq samples.**

(a) Overview of sample types and growth conditions. EGM2 media is optimized for growth of endothelial cells.

(b) Example of FACS result for isolation of SD2 GSC from a dissociated cell suspension of a brain carrying orthotopic tumor transplant. GBM cells were gated for positive staining with a human-specific anti-HLA antibody (HLA^+^). DAPI dye was used to stain dead cells.

(c) qRT-PCR analysis of expression of the endothelial cell (EC) marker gene *CDH5* in GSC isolated from different culture conditions demonstrate purity of FACS isolation from 3D GAPE culture.

**
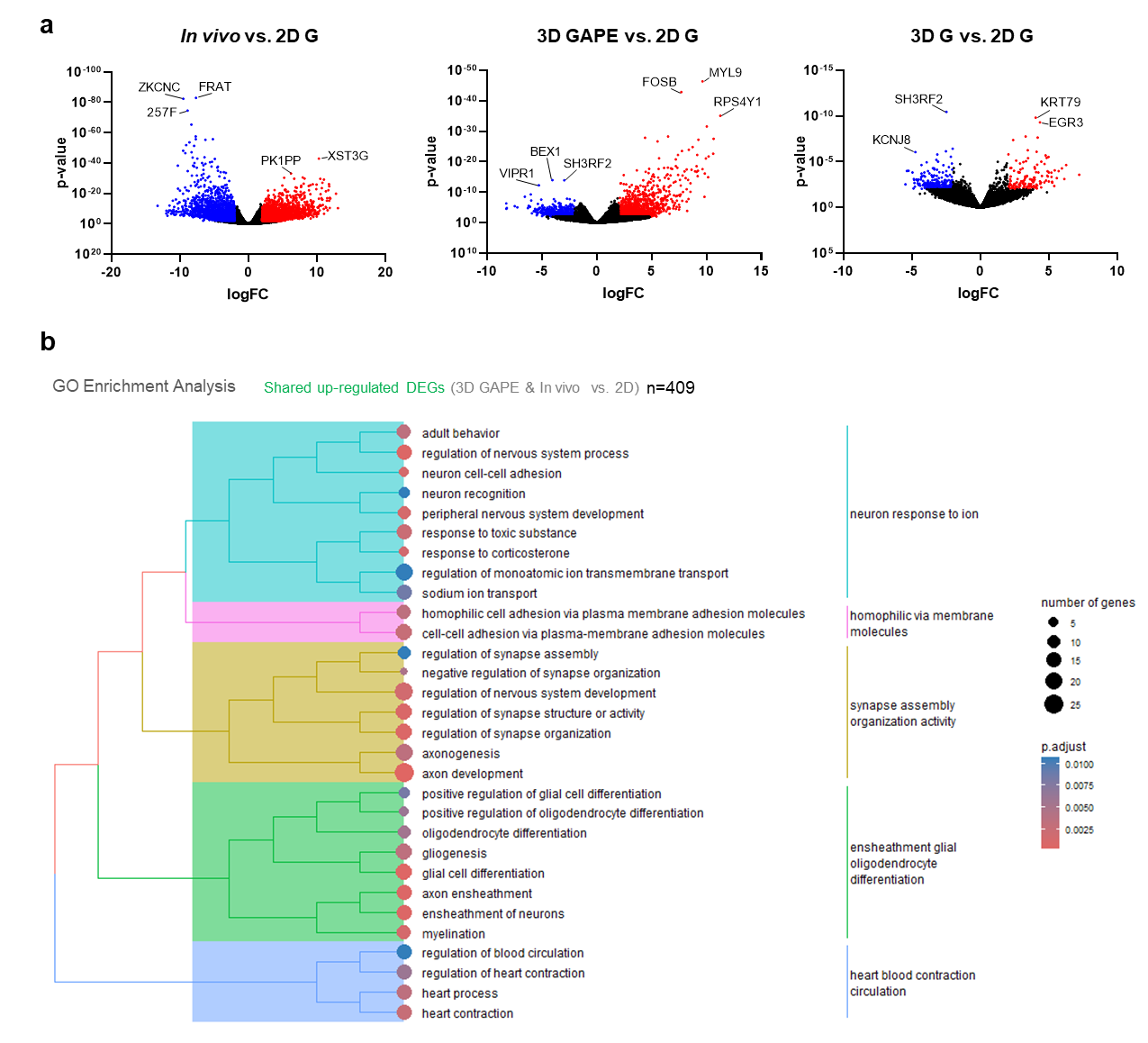
**

**Figure S4. Vulcano plot representations of differentially expressed genes (DEGs).**

(a) Vulcano plot representations of differentially expressed genes (DEGs) from comparisons of different culture conditions and *in vivo* condition. Cut-offs are *P* < 0.01 and |log2 fold-change| >2.

(b) Gene ontology (GO) enrichment analysis of upregulated shared DEGs of *in vivo* and 3D GAPE GSCs vs. 2D.

**Video S1. 3D reconstruction of the vascular niche demonstrating lumen formation and astrocyte endfeet interaction with the vasculature.**

**Video S2. 3D reconstruction of the 3D GAPE model showing lumen formation and SD3 GSCs positioned near the vasculature.**

**Table S1. Quantification of total vessel length across different seeding conditions.**

Total vessel length was measured for each condition. Statistical analysis was performed using one-way ANOVA with Tukey's post hoc test, comparing all conditions to the AC6-PC6 condition (6 × 10⁶ cells/mL). n = 4–12 per condition. Data are presented as mean ± SD. ^*^ *P* ≤ 0.05, ^**^ *P* ≤ 0.01, ^***^ *P* ≤ 0.001, ^****^ *P* ≤ 0.0001.

| d7 | | AC (×10^6^ cells/mL) | | |
| --- | --- | --- | --- | --- |
|  |  | 1.2 | 3 | 6 |
| PC (×10^6^ cells/mL) | 1.2 | 4823±354.8^***^ | 5635±365 | 5678±325.5 |
|  | 3 | 4925±117.4^***^ | 5610±122.1 | 5318±325.7^*^ |
|  | 6 | 3470±468.8^****^ | 4345±485.3^****^ | 5788±231.2 |
| d14 | | AC (×10^6^ cells/mL) | | |
|  |  | 1.2 | 3 | 6 |
| PC (×10^6^ cells/mL) | 1.2 | 5900±273.7 | 5874±225.2 | 6204±299 |
|  | 3 | 6416±162.3 | 5968±354.2 | 6082±237 |
|  | 6 | 5045±670.8^****^ | 5542±421.5^**^ | 6382±254.4 |
| d21 | | AC (×10^6^ cells/mL) | | |
|  |  | 1.2 | 3 | 6 |
| PC (×10^6^ cells/mL) | 1.2 | 5565±611.2^****^ | 6009±119.8 | 6280±294 |
|  | 3 | 6194±260.6 | 6183±231.9 | 6127±189.9 |
|  | 6 | 5549±148.6^****^ | 5800±544.9^**^ | 6504±203.6 |

**Table S2. Quantification of vessel area across different seeding conditions.**

Vessel area was measured to assess the effect of varying pericyte and astrocyte densities. Statistical analysis was conducted using one-way ANOVA with Tukey's post hoc test, comparing all conditions to AC6-PC6 (6 × 10⁶ cells/mL). n = 4–12 per condition. Data are presented as mean ± SD. ^*^ *P* ≤ 0.05, ^**^ *P* ≤ 0.01, ^***^ *P* ≤ 0.001, ^****^ *P* ≤ 0.0001.

| d7 | | AC (×10^6^ cells/mL) | | |
| --- | --- | --- | --- | --- |
|  |  | 1.2 | 3 | 6 |
| PC (×10^6^ cells/mL) | 1.2 | 55233±721.7 | 62050±396.3^****^ | 53696±948.7 |
|  | 3 | 55178±1435 | 60027±393.1^**^ | 52274±2105^*^ |
|  | 6 | 45713±3911^****^ | 49815±2831^***^ | 55258±2011 |
| d14 | | AC (×10^6^ cells/mL) | | |
|  |  | 1.2 | 3 | 6 |
| PC (×10^6^ cells/mL) | 1.2 | 56250±699.2 | 58370±746.6 | 56907±2085 |
|  | 3 | 56490±943.5 | 57543±4398 | 55383±2765 |
|  | 6 | 48953±2812^****^ | 51948±3455^**^ | 58682±1663 |
| d21 | | AC (×10^6^ cells/mL) | | |
|  |  | 1.2 | 3 | 6 |
| PC (×10^6^ cells/mL) | 1.2 | 52402±1881^****^ | 59385±1096 | 58394±1280^*^ |
|  | 3 | 57831±1287^*^ | 57874±3418^**^ | 56171±2963^***^ |
|  | 6 | 47625±2285^****^ | 46373±2910^****^ | 62121±2022 |

**Table S3. Quantification of junction density across different seeding conditions.**

Junction density was analyzed to evaluate vascular network connectivity under different cell seeding conditions. Statistical analysis was performed using one-way ANOVA with Tukey's post hoc test, comparing all conditions to AC6-PC6 (6 × 10⁶ cells/mL). n = 4–12 per condition. Data are presented as mean ± SD. ^*^ *P* ≤ 0.05, ^**^ *P* ≤ 0.01, ^***^ *P* ≤ 0.001, ^****^ *P* ≤ 0.0001.

| d7 | | AC (×10^6^ cells/mL) | | |
| --- | --- | --- | --- | --- |
|  |  | 1.2 | 3 | 6 |
| PC (×10^6^ cells/mL) | 1.2 | 27±9.122 | 54.74±11.35 | 5.355±0.5983^**^ |
|  | 3 | 47.1±19.01 | 36.26±11.87 | 15.82±4.905 |
|  | 6 | 33.31±16.11 | 22.8±13.67 | 38.78±11.6 |
| d14 | | AC (×10^6^ cells/mL) | | |
|  |  | 1.2 | 3 | 6 |
| PC (×10^6^ cells/mL) | 1.2 | 23.81±10.85^*^ | 40.89±15.93 | 14.49±12.22^**^ |
|  | 3 | 27.18±8.714^*^ | 23.71±17.57^***^ | 19.47±8.337^**^ |
|  | 6 | 30.28±22.15^*^ | 26.16±15.42^**^ | 55.7±17.8 |
| d21 | | AC (×10^6^ cells/mL) | | |
|  |  | 1.2 | 3 | 6 |
| PC (×10^6^ cells/mL) | 1.2 | 23.89±6.807^****^ | 34.93±9.663^****^ | 8.975±3.668^****^ |
|  | 3 | 41.59±4.826^***^ | 44.39±23.07^****^ | 13.68±4.711^****^ |
|  | 6 | 48.75±15.29^****^ | 42.7±21.36^****^ | 100.2±34.92 |

**Table S4. Categorization of shared differentially expressed genes (DEGs) in 3D GAPE and *in vivo* conditions based on their functional roles in neural processes.**

| **Category** | **Genes** |
| --- | --- |
| Axon Development | UNC5A[1], MGARP, MT3, KIAA1755, NCAM2, CSPG5, EGR2[2], NR4A2[3], S100B[4, 5], DOBL, ARK2C |
| Ensheathment of Neurons, Glial Cell Differentiation, and/or Axon Ensheathment | NTRK3[6], LAMC3, SOX8, ERBB3[7-9], OLIG1[10, 11], LGI4, TLR2[12], OLIG2[10, 11, 13], KCNJ10, CXCR4[14-17], ADGRG6, CLDN11, MPZ, GAL3ST1 |
| Regulation of Nervous System Processes | CACNG4[18], GRIN2D, AVPR1A, NOS3, IGSF11, OPRD1, DLGAP1[19, 20], CACNG5, EDNRB |
| Genes associated with multiple categories | CSPG5, MT3, PLP1, PTPRZ1, MBP, EGR2, IL33, LGI4, TLR2, OLIG2, CXCR4, CLDN11, MPZ, GAL3ST1, NLGN3, NRXN1 |

**REFERENCES**

[1] W. Qian, L. Zhang, F. Zhang, J. Ye, Z. Wan, H. Chen, C. Luo, *Discover Oncology* **2024**, *15* (1), 297.

[2] W. Li, M. Wang, W. Ma, P. Liu, M. Zhang, J. He, Y. Cui, *CNS Neuroscience & Therapeutics* **2023**, *29* (8), 2292.

[3] K. Karki, X. Li, U.-H. Jin, K. Mohankumar, M. Zarei, S. K. Michelhaugh, S. Mittal, R. Tjalkens, S. Safe, *Journal of Neuro-oncology* **2020**, *146*, 25.

[4] G. E. Davey, P. Murmann, C. W. Heizmann, *Journal of Biological Chemistry* **2001**, *276* (33), 30819.

[5] H. Wang, L. Zhang, I. Y. Zhang, X. Chen, A. Da Fonseca, S. Wu, H. Ren, S. Badie, S. Sadeghi, M. Ouyang, *Clinical Cancer Research* **2013**, *19* (14), 3764.

[6] D. König, J. Hench, S. Frank, L. Dima, I. Bratic Hench, H. Läubli, *Pharmacology* **2022**, *107* (7-8), 433.

[7] E. Carrasco-García, M. Saceda, S. Grasso, L. Rocamora-Reverte, M. Conde, Á. Gómez-Martínez, P. García-Morales, J. A. Ferragut, I. Martínez-Lacaci, *Experimental cell research* **2011**, *317* (10), 1476.

[8] V. Duhem-Tonnelle, I. Bièche, S. Vacher, A. Loyens, C.-A. Maurage, F. Collier, M. Baroncini, S. Blond, V. Prevot, A. Sharif, *Journal of Neuropathology & Experimental Neurology* **2010**, *69* (6), 606.

[9] M. Tagliaferro, P. Rosa, G. C. Bellenchi, D. Bastianelli, R. Trotta, C. Tito, F. Fazi, A. Calogero, D. Ponti, *BMC Molecular and Cell Biology* **2022**, *23* (1), 13.

[10] J. I. Szu, I. F. Tsigelny, A. Wojcinski, S. Kesari, *Frontiers in Neuroscience* **2023**, *17*, 1129434.

[11] L. Aguirre-Cruz, K. Mokhtari, K. Hoang-Xuan, Y. Marie, E. Criniere, S. Taillibert, M. Lopes, J.-Y. Delattre, M. Sanson, *Journal of neuro-oncology* **2004**, *67*, 265.

[12] J. H. Park, I. Kang, H. K. Lee, *Frontiers in Immunology* **2022**, *13*, 1044544.

[13] I. F. Tsigelny, V. L. Kouznetsova, N. Lian, S. Kesari, *Oncotarget* **2016**, *7* (33), 53074.

[14] M. Ehtesham, J. Winston, P. Kabos, R. Thompson, *Oncogene* **2006**, *25* (19), 2801.

[15] F. Gagliardi, A. Narayanan, M. Reni, A. Franzin, E. Mazza, N. Boari, M. Bailo, P. Zordan, P. Mortini, *Glia* **2014**, *62* (7), 1015.

[16] V. N. Yadav, D. Zamler, G. J. Baker, P. Kadiyala, A. Erdreich-Epstein, A. C. DeCarvalho, T. Mikkelsen, M. G. Castro, P. R. Lowenstein, *Oncotarget* **2016**, *7* (50), 83701, <https://doi.org/10.18632/oncotarget.13295>.

[17] Y. Zhou, P. H. Larsen, C. Hao, V. W. Yong, *Journal of Biological Chemistry* **2002**, *277* (51), 49481.

[18] M.-E. Halatsch, S. Loew, K. Mursch, T. Hielscher, U. Schmidt, A. Unterberg, V. I. Vougioukas, F. Feuerhake, *Journal of neurosurgery* **2009**, *111* (2), 211.

[19] Y. Li, W. Li, X. Zeng, X. Tang, S. Zhang, F. Zhong, X. Peng, Y. Zhong, T. J. Rosol, X. Deng, *Oncogene* **2019**, *38* (47), 7234.

[20] W. Miao, N. Li, B. Gu, G. Yi, Z. Su, H. Cheng, *The journal of biochemistry* **2020**, *167* (4), 411.
